# Regulatory role of RNA-binding proteins in microRNA biogenesis

**DOI:** 10.3389/fmolb.2024.1374843

**Published:** 2024-03-19

**Authors:** Claire Hynes, Pavan Kumar Kakumani

**Affiliations:** Department of Biochemistry, Memorial University of Newfoundland, St. John’s, NL, Canada

**Keywords:** RBPs, miRNAs, processing, biogenesis, gene regulation

## Abstract

MicroRNAs (miRNAs) are small non-coding RNAs that silence gene expression through their interaction with complementary sequences in the 3′ untranslated regions (UTR) of target mRNAs. miRNAs undergo a series of steps during their processing and maturation, which are tightly regulated to fine-tune their abundance and ability to function in post-transcriptional gene silencing. miRNA biogenesis typically involves core catalytic proteins, namely, Drosha and Dicer, and several other RNA-binding proteins (RBPs) that recognize and interact with miRNA precursors and/or their intermediates, and mature miRNAs along with their interacting proteins. The series of RNA-protein and protein-protein interactions are critical to maintaining miRNA expression levels and their function, underlying a variety of cellular processes. Throughout this article, we review RBPs that play a role in miRNA biogenesis and focus on their association with components of the miRNA pathway with functional consequences in the processing and generation of mature miRNAs.

## 1 Introduction

miRNAs are small non-coding RNAs of approximately 21–23 nucleotides (nt) in length (Berezikov et al., 2006). These small RNAs are produced through a series of biogenesis steps beginning in the nucleus and ending in the cytosol. The process begins with a primary miRNA (pri-miRNA) transcript which is cleaved to produce precursor miRNA (pre-miRNA), followed by another cleavage step, generating mature miRNA (Davis-Dusenbery and Hata, 2010). Mature miRNAs are associated with Argonaute (AGO) proteins which facilitate their function in gene silencing at the post-transcriptional level. The miRNA guides the complex to bind complementary mRNA targets while the AGO protein recruits various other proteins that promote translational inhibition and/or mRNA decay (Gebert and MacRae, 2019). Thus, miRNA biogenesis must be tightly regulated at many levels to ensure accuracy in the regulation of specific gene expression.

RNA-binding proteins (RBPs) are critical for miRNA biogenesis and they often have conserved RNA binding domains that recognize certain sequence elements or secondary structures in the RNA (Lunde et al., 2007; Lukong et al., 2008). For example, the double-stranded RNA binding domain (dsRBD) (Kwon et al., 2016), zinc finger domain, cold shock domain (CSD), RNA recognition motif (RRM), and KH (K homology) domain (Castilla-Llorente et al., 2013). RBPs may also consist of intrinsically disordered regions that interact with RNA (Basu and Bahadur, 2016). RBPs fine-tune miRNA expression through various biogenesis steps: nuclear processing, nuclear export, cytosolic processing and loading of miRNAs into RNA-induced silencing complex (RISC). Furthermore, various RBPs interact with miRISC to regulate miRNA function. Essentially, RBPs are the primary means to alter miRNA abundance and function (van Kouwenhove et al., 2011).

## 2 Overview of canonical miRNA biogenesis

**FIGURE 1:**
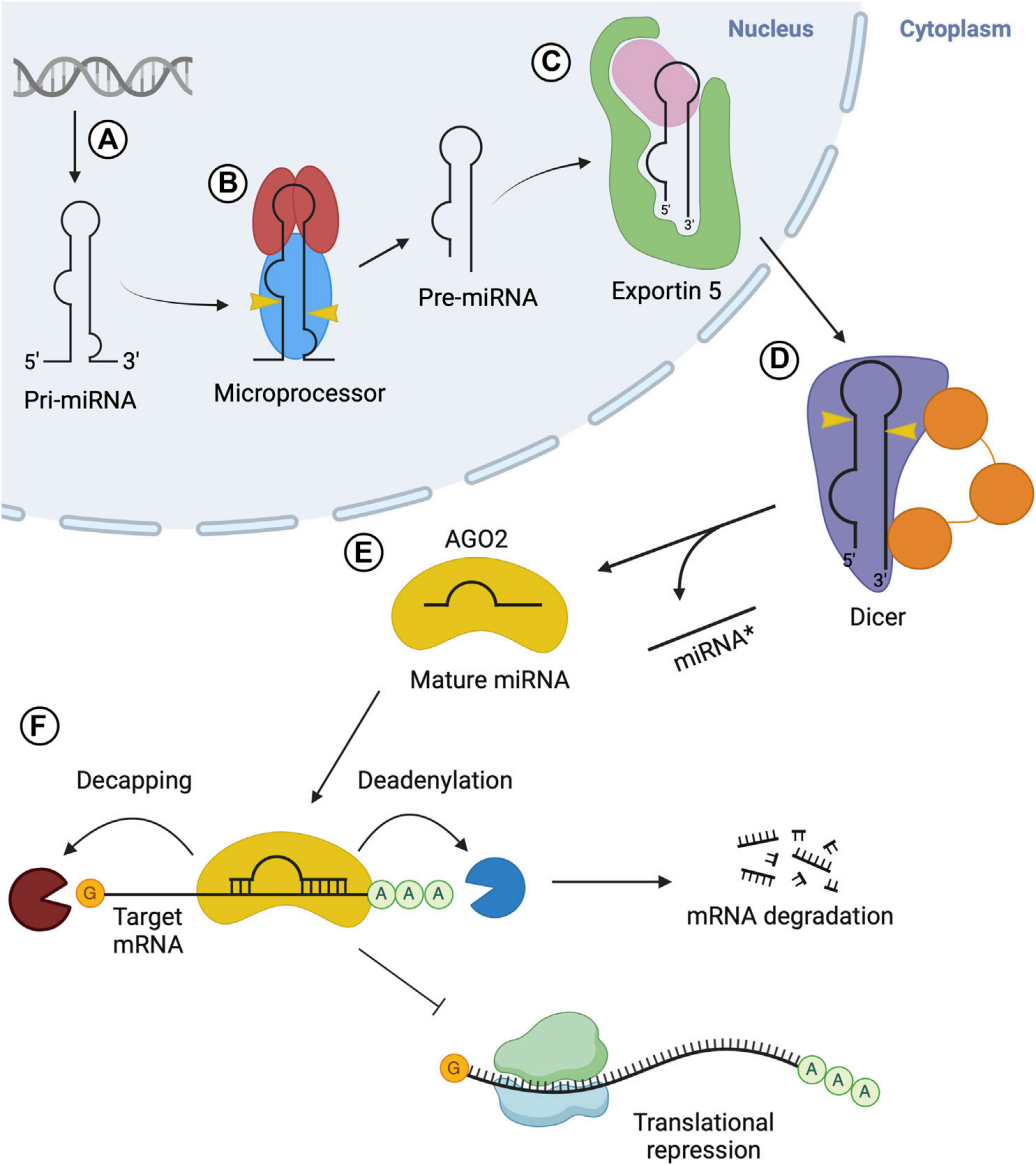
Overview of microRNA biogenesis. **(A)** Transcription via RNA Pol II produces pri-miRNA. **(B)** The microprocessor complex composed of Drosha (blue) and two DGCR8 (red) molecules cleaves pri-miRNA, resulting in pre-miRNA. **(C)** Exportin 5 (green) carries out nuclear export of the pre-miRNA. **(D)** Dicer (purple) cleaves the pre-miRNA in the cytosol, generating a mature miRNA duplex. **(E)** The miRNA duplex is transferred from Dicer to an AGO protein, where the passenger strand (miRNA* or 3p) is released while the guide strand (5p) remains associated with AGO, forming the miRISC. **(F)** RISC is recruited to target mRNA via interactions with the guide strand and delivers gene silencing by promoting deadenylation and/or decapping of target mRNA, or via translational inhibition of the mRNA.

Canonical miRNA biogenesis is initiated through the transcription of miRNA genes, which is carried out by RNA polymerase II (Lee et al., 2004) (Figure 1A). Several miRNAs tend to be encoded adjacently in the transcript and are transcribed in clusters known as polycistronic transcripts. These transcripts are then subsequently processed into individual miRNAs. miRNAs are often encoded in introns of protein-coding regions, but may also be encoded in exons (Lee et al., 2002; Carthew and Sontheimer, 2009). Transcription of miRNA genes results in the immature miRNA transcript, pri-miRNA, which must undergo a series of maturation steps to produce a mature miRNA to exert regulatory effects. The pri-miRNA is a relatively long transcript, often 1–10 kb in length (Saini et al., 2008), containing a stem-loop structure in which the mature miRNA sequence is located (Lee et al., 2002). The pri-miRNA molecule is processed into pre-miRNA in the nucleus via the nuclear protein complex known as the microprocessor (Figure 1B). The microprocessor consists of Drosha, which retains the catalytic activity required for this step, two DiGeorge syndrome critical region 8 (DGCR8) molecules and other auxiliary factors including DDX5/p68 and DDX17/p27 (Gregory et al., 2004; Han et al., 2004). Drosha is an RNase III enzyme which cleaves the long precursor transcript into a shorter, single hairpin structure consisting of approximately 65 nucleotides (nt), termed pre-miRNA (Lee et al., 2003).

Following nuclear processing, the resulting pre-miRNA is exported to the cytoplasm to undergo processing into mature miRNA. This nuclear export step is carried out by the export receptor, exportin 5 (Exp5), through the formation of an export complex with RAN-GTP and the pre-miRNA (Figure 1C). The complex undergoes translocation through the nuclear pore complex, resulting in the release of the pre-miRNA in the cytosol (Bohnsack et al., 2004; Lund et al., 2004). In the cytoplasm, pre-miRNA is processed into a mature miRNA molecule, through a cleavage step carried out by Dicer, an RNase type III enzyme (Zhang et al., 2004) (Figure 1D). This endoribonuclease cleaves a double-stranded segment of 20–25 nt in length from the stem structure, which is known as mature miRNA (Grishok et al., 2001; Ketting et al., 2001). In humans, Dicer often interacts with the trans-activation-responsive (TAR) RNA-binding protein (TRBP) RBP to enhance its activity (Chendrimada et al., 2005; Haase et al., 2005). The double-stranded mature miRNA molecule generated by Dicer is loaded onto Argonaute (AGO) family proteins forming the RISC (Figure 1E). This process is termed RISC loading. Next, one strand is selected from the miRNA duplex, termed as the guide strand, while the other (passenger strand) dissociates from the complex (Kawamata and Tomari, 2010; Kobayashi and Tomari, 2016). Once strand selection is complete, the AGO protein and guide strand complex can dissociate from Dicer. The RISC complex then functions in gene silencing (Figure 1F). The miRNA sequence acts to guide RISC to complementary target mRNA sequences in the 3′untranslated regions (UTR) (Treiber et al., 2019).

## 3 RBPs in microprocessor-mediated pri-miRNA processing

The key components of the microprocessor include one Drosha and two DGCR8 molecules (Nguyen et al., 2015; Herbert et al., 2016). DGCR8 forms a dimer, which is stimulated through the binding of heme (Weitz et al., 2014). The heme-bound dimer forms a trimer with Drosha, which carries out the processing of pri-miRNA. Inducing the active conformation of DGCR8 may be, in part, attributed to the binding of heme (Quick-Cleveland et al., 2014). The Drosha protein contains an unstructured N-terminus (critical for nuclear localization), a central domain, two catalytic RNase III domains and C-terminal double-stranded RNA binding domains (dsRBDs) (Kwon et al., 2016). Upon formation of the heterotrimer between Drosha and DGCR8, the two RNase III domains that constitute the catalytic center of Drosha bind one of the DGCR8 helices (Kwon et al., 2016). The DGCR8 molecules also contain dsRBDs. The dsRBDs of Drosha and DGCR8 are essential for the recognition and binding of pri-miRNAs, through their conserved αβββα motifs (Kharrat et al., 1995). Specific secondary structures characteristic of this motif, including the N-terminal α-helix, loop between β-strand one and 2, and the C-terminal α-helix recognize features of RNA, such as minor and major grooves and the 2′OH group of the ribose in RNA (Ryter and Schultz, 1998; Partin et al., 2020).

Pri-miRNA molecules contain a critical stem-loop structure with flanking regions at both 3′and 5′ends (Altuvia et al., 2005). Unique features of pri-miRNA molecules are essential for recruiting the microprocessor for nuclear processing. These features include the characteristic stem-loop as well as several sequence features (Auyeung et al., 2013; Fang and Bartel, 2015). The dsRBD of Drosha and DGCR8 are responsible for the recognition of these sequence elements in pri-miRNA. In the heterotrimeric complex, Drosha binds at the stem-flank junction (basal junction), through the recognition of conserved UG motifs via the central domain. Meanwhile, DGCR8 binds the terminal loop of the hairpin and recognizes the conserved UGU motif which is essential for an accurate cleavage (Faller et al., 2010; Nguyen et al., 2015). Following the binding of the microprocessor to specific pri-miRNA elements, processing to pre-miRNA will be carried out through the catalytic activity of Drosha’s two RNase III domains. Drosha acts as a ‘molecular ruler’ to define the distance from the cleavage site to the basal junction (Blaszczyk et al., 2001; Kwon et al., 2016). This results in cleavage at 11 bp from the basal junction, generating a pre-miRNA molecule with a 2 nt overhang at the 3′end (Blaszczyk et al., 2001).

Regulation of proteins in the microprocessor complex serves as a means to control miRNA expression. This is achieved through processes that regulate the amount and stability of microprocessor proteins, thus affecting pri-miRNA processing efficacy. Autoregulation between Drosha and DGCR8 helps control levels of protein available to form the functional microprocessor complex. DGCR8 has a stabilizing effect on Drosha through their protein-protein interactions, while Drosha destabilizes DGCR8 through cleavage of its mRNA (Han et al., 2009). Another prominent means of regulation is through RBPs interacting with components of the microprocessor or sequence features of pri-miRNAs. The ability of RBPs to recognize these sequence elements is critical to pri-miRNA processing efficacy and mutations in the motifs or dysregulation affect pri-miRNA processing and are evident in the development of numerous human diseases. For instance, the DDX5 (p68) and DDX17 RBPs (p72) are required for efficient pri-miRNA processing to occur (Shiohama et al., 2007; Beezhold et al., 2010). These proteins belong to the DEAD-box RNA helicase family and are believed to associate with the microprocessor complex to promote pri-miRNA processing. They are predicted to unwind RNA, in an ATP-dependent manner, to make it more accessible for cleavage by Drosha (Janknecht, 2010).

**FIGURE 2:**
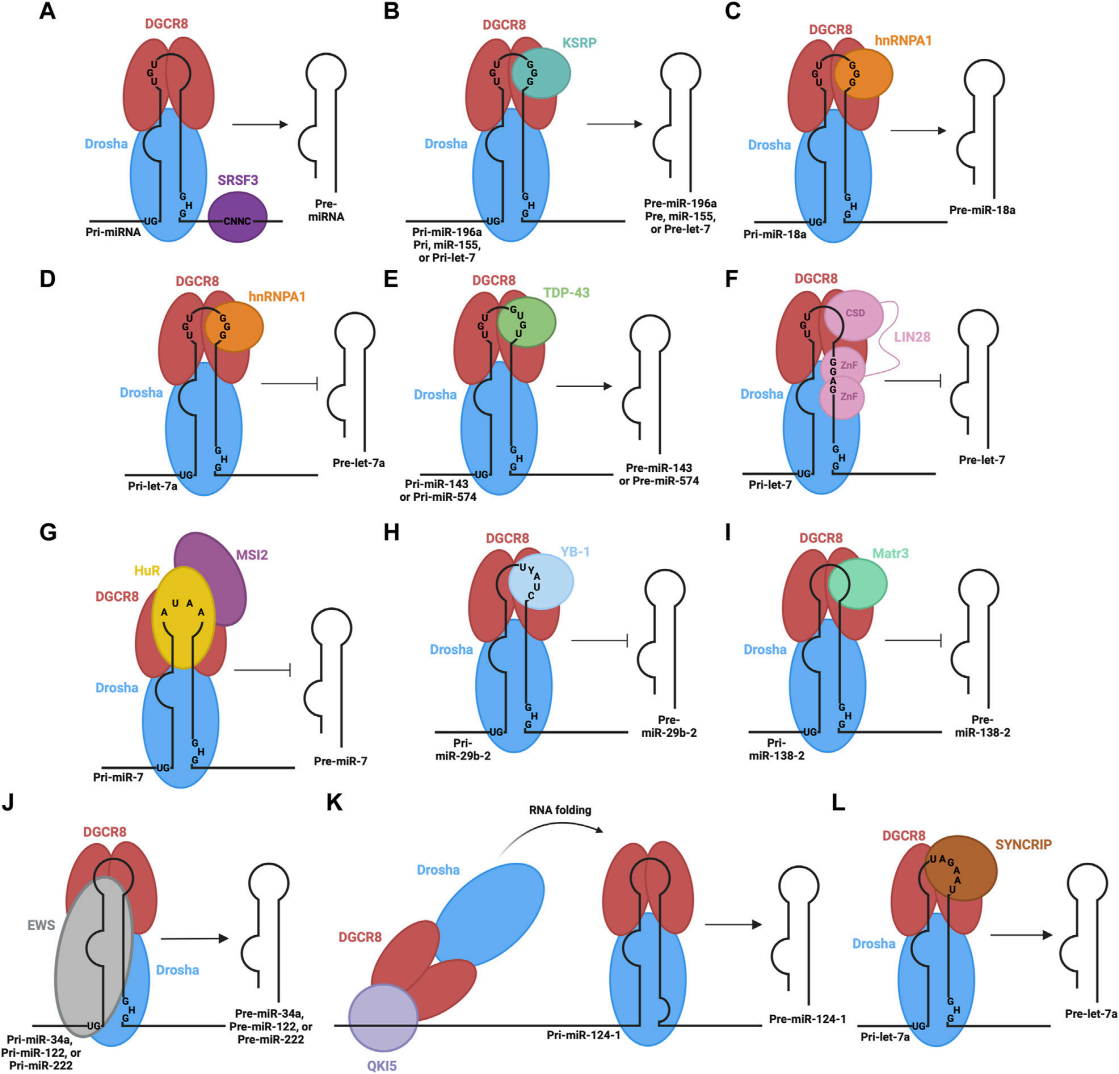
RBPs involved in the processing of pri-miRNA in the nucleus. The microprocessor complex, consisting of Drosha and DGCR8 proteins, associates with pri-miRNA through their RNA binding domains and recognition of sequence motifs. Different RBPs also associate with different sequence elements of pri-miRNA: **(A)** SRSF3 promotes microprocessor-mediated pri-miRNA cleavage, **(B)** KSRP promotes microprocessor-mediated cleavage of pri-miR-196a, pri-miR-155, and pri-let-7, **(C)** hnRNPA1 promotes microprocessor-mediated cleavage of pri-miR-18a, **(D)** hnRNPA1 hinders microprocessor-mediated cleavage of pri-let-7a, **(E)** TDP-43 promotes microprocessor-mediated cleavage of pri-miR-143 and pri-miR-574, **(F)** LIN28B inhibits microprocessor-mediated cleavage of pri-let-7, **(G)** HuR and MSI2 inhibit microprocessor-mediated cleavage of pri-miR-7, **(H)** YB-1 inhibits microprocessor-mediated cleavage of pri-miR-29b-2, **(I)** Matr3 inhibits microprocessor-mediated cleavage of pri-miR-138-2, **(J)** EWS promotes microprocessor-mediated cleavage of pri-miR-34a, pri-miR-122, and pri-miR-222, **(K)** QKI5 promotes microprocessor-mediated cleavage of pri-miR-124-1 through recruitment to a distal QRE sequence and subsequent RNA folding to bring the microprocessor to the appropriate location, **(L)** SYNCRIP promotes microprocessor-mediated cleavage of pri-let-7a.

In addition to the critical sequence or structural elements recognized by the Drosha-DGCR8 complex, there are other sequence features, such as the CNNC motif in the 3′flanking region, which are important to help stimulate enzymatic cleavage of the hairpin. For instance, the Serine/Arginine-rich splicing factor (SRSF3) specifically recognizes the CNNC motif to enhance the processing of pri-miRNA into pre-miRNA by the microprocessor (Auyeung et al., 2013) (Figure 2A). Similarly, other RBPs bind to the terminal loop of specific pri-miRNAs to either promote or impede processing. For instance, K-homology splicing regulator protein (KSRP) is another RBP that interacts with a specific set of miRNAs as well as the Drosha complex to promote biogenesis (Castilla-Llorente et al., 2013). KSRP interacts with the G-rich regions (stretches of at least 3 G’s) of the terminal loop of pri-miRNAs belonging to the let-7 family, miR-196a and miR-155 through its KH domains, facilitating the recruitment or positioning of processing factors (Ruggiero et al., 2009; Trabucchi et al., 2009) (Figure 2B).

Heterogeneous nuclear ribonucleoprotein A1 (hnRNPA1) is yet another RBP involved in pri-miRNA processing. It recognizes G-rich sequences in the terminal loop of pri-miRNAs through its RNA recognition motifs (RRM) (Castilla-Llorente et al., 2013). Specifically, interaction in this manner positively regulates pri-miR-18a and negatively regulates pri-miR-let7a (Guil and Cáceres, 2007; Michlewski and Cáceres, 2010). In the case of pri-miR-18a, hnRNPA1 binds the terminal loop, creating a more optimal cleavage site for Drosha, facilitating increased miR-18a production (Michlewski et al., 2008) (Figure 2C). Alternatively, hnRNPA1 binds the terminal loop of pri-let7a, blocking Drosha-mediated cleavage (Michlewski and Cáceres, 2010) (Figure 2D). The binding sites on pri-let-7a for hnRNPA1 and KSRP overlap, suggesting there may be competition to carry out their antagonistic regulatory roles that result in the overall regulation of this pri-miRNA processing (Michlewski and Cáceres, 2010). Likewise, the Tar-DNA binding protein of 43 kDa (TDP-43) contains two RNA recognition motifs, which are involved in the interaction with pri-miRNAs and Drosha itself, facilitating microprocessor-mediated processing (Kawahara and Mieda-Sato, 2012). TDP-43 binding helps stabilize Drosha, promoting its role in pri-miRNA cleavage (Di Carlo et al., 2013), while also binding GU-rich sequences in the terminal loops of miR-143 and miR-574 (Castilla-Llorente et al., 2013) (Figure 2E). In contrast, abnormal cell lineage factor 28 (LIN28B) binds to the terminal loop of pri-let-7 to suppress processing by the microprocessor (Michlewski et al., 2008; Piskounova et al., 2011). LIN28 contains two CCHC-type zinc finger domains and a cold shock domain (CSD), which are RNA binding domains that interact with the terminal loop of the let-7 family pri-miRNAs (Mayr and Heinemann, 2013). The CSD does not display significant sequence specificity but binds a region in the terminal loop that induces a change in the secondary structure (Mayr et al., 2012). This structural change allows the zinc finger domains to recognize and bind a specific GGAG motif (Heo et al., 2009; Nam et al., 2011) and the interaction impedes Drosha-mediated processing of the pri-miRNA (Figure 2F). Other RBPs involved in the inhibition of microprocessor-mediated processing are Hu antigen R (HuR) protein and Musashi homolog 2 (MSI2) protein (Choudhury et al., 2013). HuR binds to the conserved terminal loop of pri-miR-7, containing the sequence AUAA, which assists in the recruitment and binding of MSI2. The binding of MSI2 is expected to increase the rigidity of the pri-miRNA stem-loop structure, leading to the inhibition of cleavage by the microprocessor (Figure 2G). Additionally, the Human Y-box binding protein (YB-1) blocks the biogenesis of miR-29b-2 through binding to the UYAUC (where Y represents A or C) binding motif in the terminal loop of the pri-miRNA, inhibiting Drosha-mediated cleavage (Figure 2H) (Wu et al., 2015). This is expected to occur through two proposed mechanisms. YB-1 binding may alter the structure of the pri-miRNA, through its chaperon activity, so that it is not a suitable substrate for cleavage (Skabkin et al., 2001) or YB-1 may form oligomeric complexes with RNA that could block the microprocessor from accessing the pri-miRNA (Skabkin et al., 2004). Similarly, Matrin-3 (Matr3), a nuclear matrix protein, binds the terminal loop of pri/pre-miR-138-2 in the nucleus through its ZNF2 domain (Figure 2I) (Weiss et al., 2019). This may hinder microprocessor-mediated processing or nuclear export.

Other RBPs may rely on stem flanking sequences in pri-miRNA for binding and recruitment. For example, Ewing sarcoma protein (EWS) is expected to bind stem-loop flanking regions in a wide variety of pri-miRNAs, including miR-34a, miR-122 and miR-222 (Figure 2J) (Ouyang et al., 2017). Importantly, the terminal loop also contributes to the binding of EWS. It is also expected that EWS can recruit the microprocessor to chromatin, co-transcriptionally, so it is likely that the microprocessor can be loaded directly to certain pri-miRNAs from chromatin via EWS-mediated interactions (Ouyang et al., 2017). Furthermore, a recent study shows how distal elements in the pri-miRNA transcript may be involved in recruiting and regulating the microprocessor step (Wang et al., 2017). Here, the QKI5 RBP recognizes the QKI response element (QRE), approximately 300 nt upstream of the stem-loop in pri-miR-124-1. QKI5 then recruits the microprocessor through interaction with the double-stranded RNA binding motifs in DGCR8. Next, a spatial RNA-RNA interaction between complementary regions near the QRE and stem-loop of pri-miR-124-1 brings the recruited microprocessor into the proximity of the stem-loop where processing can proceed (Figure 2K). This regulatory process is important in erythropoiesis. Collectively, these studies indicate that RBPs can alter the outcome of pri-miRNA processing through their binding of sequence and/or structural elements in pri-miRNA sequences and interaction with, or manipulation of the function of the components of microprocessor complex.

## 4 RBPs in nuclear export of pre-miRNA

Following the nuclear processing of pri-miRNAs to pre-miRNAs, the resulting pre-miRNA molecules must be exported into the cytoplasm to complete their maturation. For canonical miRNA export, this process is carried out by the Exportin five protein (Exp5) (Bohnsack et al., 2004; Lund et al., 2004). The Exp5 export protein forms a complex with RAN-GTP and the pre-miRNA and translocates through the nuclear pore complex into the cytosol. Here, GTP hydrolysis occurs via the RAN GTPase, leading to the disassembly of the export complex and the release of the pre-miRNA into the cytosol. In cases of non-canonical miRNA biogenesis, export may be facilitated by other export factors, such as exportin 1 (Xie et al., 2013).

**FIGURE 3:**
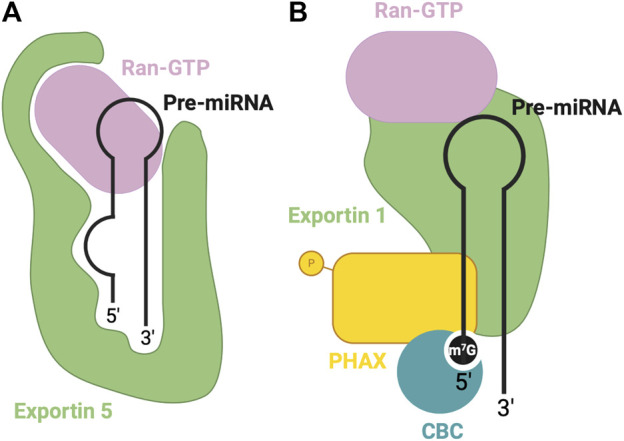
RBPs involved in nuclear export of pre-miRNA. **(A)** Canonical pre-miRNA nuclear export complex: Exportin five accommodates pre-miRNA in a deep pocket, and associates with RAN-GTP to facilitate transport through the nuclear pore complex. **(B)** Non-canonical m7G cap dependent pre-miRNA nuclear export complex: CBC recognizes the m7G cap, associated with Exportin 1-RAN-GTP through PHAX bridge.

Exportin five is a dsRNA binding protein (dsRBP) that recognizes the pre-miRNA mainly through sequence-independent ionic interactions (Okada et al., 2009). Specifically, the Exp5-RAN-GTP complex recognizes the double-stranded stem and 2-nt overhang at the 3′end of the pre-miRNA. Exp5 forms a structure reminiscent of a baseball mitt, which encapsulates the pri-miRNA, with a tunnel-like structure at the bottom of the mitt that interacts with the 2-nt overhang (Figure 3A). Not only does this interaction facilitate transport, but it also protects the miRNA from degradation (Okada et al., 2009).

Alternatively, Exportin 1-mediated miRNA export is required for the non-canonical nuclear export of pre-miRNAs with a 7-methylguanosine (m7G) cap (Xie et al., 2013). These miRNAs are transcribed by the RNA polymerase II, and a m7G cap is added to their 5′end, post-transcriptionally. The resulting transcript folds into a hairpin miRNA structure which can bypass the microprocessor biogenesis step. The miRNAs are recognized by their m7G cap, and as a result, exported by the PHAX-Exportin one pathway (Xie et al., 2013). The PHAX-Exportin one pathway has previously been established for U snRNA precursors (Ohno et al., 2000). A nuclear cap-binding complex (CBC) recognizes and interacts with the 7 mG cap (McCloskey et al., 2012). Exportin one must also associate with a RAN GTPase to facilitate export. Phosphorylated adapter RNA export protein (PHAX) associates with CBC and exportin 1, bridging these components (Ohno et al., 2000) (Figure 3B). For proper assembly and disassembly of this export machinery, PHAX must be phosphorylated in the nucleus and subsequently dephosphorylated in the cytoplasm (Figure 5B). In the cytoplasm, these Exportin 1-dependent miRNAs are processed normally by Dicer. However, strand selection is biased towards the 3p-miRNA, as the m7G cap at the 5′end may interfere with the association with the AGO2 (Ohno et al., 2000). Two miRNAs that follow this pathway are miR-320 and miR-484, which are involved in the PTEN tumor suppressor pathway (Bronisz et al., 2012; Wang et al., 2012). Interestingly, Exportin 1-dependent export may also be important during cellular quiescence, during which Exportin five is downregulated (Martinez et al., 2017), whereby Exportin 1 -dependent miRNAs are upregulated. In this case, pri-miRNAs may undergo hypermethylation via TGS1 to provide the m7G cap required for export (Martinez et al., 2017), suggesting the presence of two distinct miRNA biogenesis pathways requiring Exportin 1.

## 5 RBPs in dicer-mediated processing of pre-miRNA

Once the pre-miRNA is released into the cytoplasm, Dicer completes the maturation process of the pre-miRNA, through its RNase III activity. Cleavage of the pre-miRNA produces mature miRNA duplexes that can be loaded onto AGO proteins to carry out their regulatory role in gene silencing. The Dicer protein has an L-shaped structure and contains a PAZ (PIWI-AGO-ZWILLE) domain, helicase domain, platform domain, two RNase III domains and a dsRBD (Zhang et al., 2004). Dicer possesses its catalytic activity due to its two RNase III domains which dimerize, forming a catalytic center (Zhang et al., 2004). Upon binding a pre-miRNA, Dicer undergoes a structural rearrangement which converts it into a productive state (Taylor et al., 2013). Binding the pre-miRNA is achieved through different domains in the Dicer molecule; the helicase domain binds the terminal loop (Tsutsumi et al., 2011), a pocket in the PAZ domain anchors the 2-nt 3′overhang and a pocket in the platform domain binds the 5′phosphate (Park et al., 2011). To produce a mature miRNA of appropriate length, Dicer acts as a ‘molecular ruler’, resulting in cleavage of the terminal loop, approximately 22 nt from the 3′end (Vermeulen et al., 2005; MacRae et al., 2006). Additionally, in mammals, Dicer also measures the cleavage site 22 nt from the 5′end to which it is bound (Park et al., 2011). This results in a mature miRNA duplex, approximately 20–25 nt in length.

**FIGURE 4:**
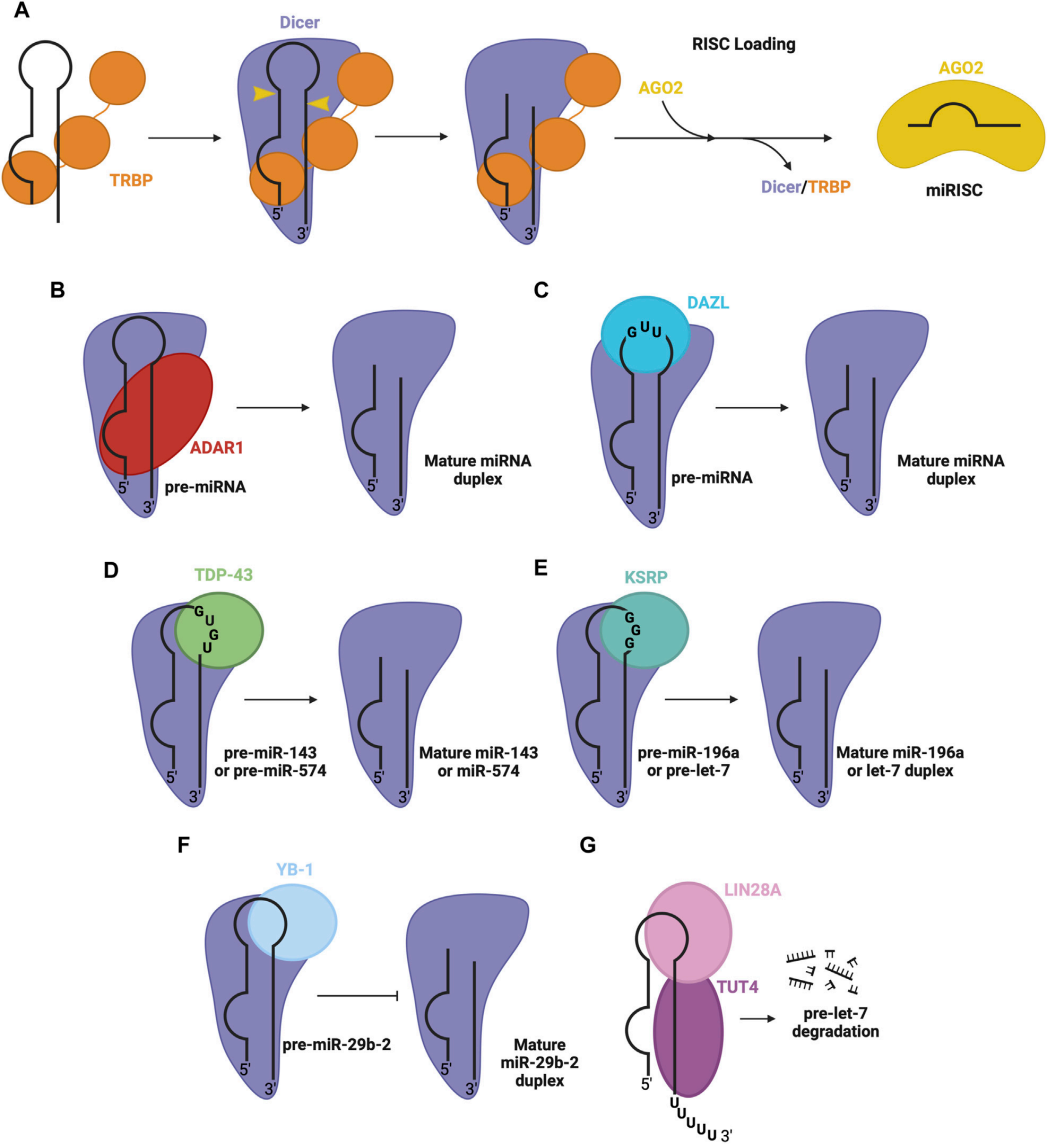
RBPs involved in pre-miRNA processing in the cytosol. **(A)** Dicer and TRBP associate with pre-miRNA through RNA binding domains to facilitate Dicer-mediated cleavage of pre-miRNA, to produce the mature miRNA duplex. The duplex is passed on to an AGO protein (e.g., AGO2), forming mature RISC. Other RBPs associate with Dicer and specific pre-miRNA sequence features, promoting the Dicer processing step, namely, **(B)** ADAR1 promotes Dicer-mediated cleavage of pre-miRNA, **(C)** DAZL promotes Dicer-mediated cleavage of pre-miRNA, **(D)** TDP-43 promotes Dicer-mediated cleavage of pre-miR-143 and pre-miR-574, **(E)** KSRP promotes Dicer-mediated cleavage of pre-miR-196a and pre-let7, **(F)** YB-1 inhibits Dicer-mediated cleavage of pre-miR-29b-2 **(G)** LIN28A inhibits Dicer-mediated processing of pre-let-7. LIN28A also recruits uridylyl transferase, TUT4, promoting pre-miRNA degradation.

To achieve efficient processing of the pre-miRNA into mature miRNA duplex, Dicer co-operates with TRBP, a dsRBP (Chendrimada et al., 2005; Haase et al., 2005). TRBP contains three dsRBDs, two of which bind pre-miRNA, while the third interacts with the DExD/H-box helicase domain of Dicer, anchoring the proteins together and stimulating Dicer (Yoshida et al., 2021) (Figure 4A). TRBP binds pre-miRNA with a preference for specific secondary structures, particularly, pre-miRNA stem regions with tight base pairing (Takahashi et al., 2018). The cooperation between these proteins promotes Dicer activity and helps produce a mature miRNA of appropriate length, although it is not essential for Dicer’s proper functioning (Chakravarthy et al., 2010). PACT is another co-factor which associates with Dicer to mediate its function similarly to TRBP (Lee et al., 2013).

Beyond TRBP, several other RBPs regulate the Dicer processing step. One prominent example is adenosine deaminase acting on RNA 1 (ADAR1), a dsRBP that complexes with Dicer’s DExD/H-box helicase domain to promote efficient miRNA processing and RISC loading (Figure 4B) (Ota et al., 2013). Another RBP that interacts with the Dicer complex is deleted in azoospermia-like (DAZL), through recognition of GUU sequence motifs in many pre-miRNA via its RRM (Figure 4C) (Yan et al., 2022). This interaction generally enhances Dicer’s cleavage activity, promoting the biogenesis of mature miRNA. Additionally, RBPs implicated in the regulation at the Drosha level, including TDP-43 (Kawahara and Mieda-Sato, 2012), KSRP (Trabucchi et al., 2009) and YB-1 (Wu et al., 2015), are also involved in the regulation of Dicer activity through interactions with the terminal loop of pre-miRNA and Dicer in the cytosol. Here, TDP-43 recognizes GU-rich sequences in the terminal loop of pre-miR-143 and pre-miR-574 (Castilla-Llorente et al., 2013) (Figure 4D). Similarly, KSRP recognizes G-rich regions in the terminal loop of let-7 family miRNA and miR-196a (Trabucchi et al., 2009) (Figure 4E). YB-1 binds the terminal loop of pre-miR-29b-2, which inhibits processing via Dicer (Figure 4F) (Wu et al., 2015). Furthermore, another isoform of LIN28, LIN28A, can function at the Dicer level, by associating with the terminal loop of pre-let-7 in the cytoplasm, inhibiting Dicer processing through changing the secondary structure of the miRNA (Lightfoot et al., 2011; Nam et al., 2011; Piskounova et al., 2011). LIN28A also recruits TUT4, a uridylyl transferase, which destabilizes and promotes the degradation of the miRNA (Heo et al., 2008; Heo et al., 2009) (Figure 4G).

## 6 RBPs in RISC formation and miRNA function

Once Dicer has produced a miRNA duplex in the cytoplasm, it is passed on to AGO family proteins and a single strand (guide) is selected to function as the mature miRNA, while the other (passenger strand) dissociates. The result is an RNA-induced silencing complex (RISC), which is the functional entity in miRNA-mediated post-transcriptional target repression (Kobayashi and Tomari, 2016) (Figure 1E). Interestingly, there are four AGO proteins in humans, which are all capable of miRNA-directed gene silencing (Azuma-Mukai et al., 2008). AGO proteins are composed of the N-terminal domain, PAZ domain, MID domain and PIWI domain (Ipsaro and Joshua-Tor, 2015). They take on a bilobal conformation in which the N-terminal and PAZ domains form the N-terminal lobe, while the MID and PIWI domains form the C-terminal lobe (Ha and Kim, 2014). The PAZ and MID domains are important for RNA binding, while the PIWI domain has endonuclease activity (Song et al., 2004). However, among the AGO proteins in humans (AGO1-4), only AGO2 has catalytic activity and is able to cleave double-stranded miRNAs (Liu et al., 2004).

During RISC loading, Dicer, TRBP and AGO proteins form a ternary complex, known as the RISC loading complex (RLC), which facilitates the transfer of the miRNA duplex to the AGO protein (Gregory et al., 2005). TRBP supports the direct handover of the double-stranded miRNA from Dicer to AGO (Wang et al., 2009). The MID domain of AGO anchors the 5′phosphate group of the miRNA in a binding pocket, while the PAZ domain recognizes the 2 nt 3′overhang produced by Dicer (Ma et al., 2004; Elkayam et al., 2012). Throughout the RISC loading process, AGO undergoes a series of conformational changes to facilitate the loading process; an initial ‘apo’ state whereby there is no miRNA bound, a pre-RISC state in which the miRNA duplex is bound, and a final state in which the mature miRNA is bound (Nakanishi, 2016). Heat shock protein 90 (HSP90)/heat shock cognate 70 (HSC70) chaperone complex facilitates loading of the miRNA duplex onto AGO, using ATP, creating tension in the structure of AGO to induce an open conformation (Iwasaki et al., 2010; Kawamata and Tomari, 2010).

Once the miRNA duplex has been loaded onto the AGO protein, strand selection must occur to generate mature RISC. Here, one strand is selected as the mature miRNA, known as the guide strand, while the passenger strand dissociates. First, the unwinding of the RNA duplex must occur, which is often promoted by mismatches in the guide strand (Yoda et al., 2010). Next, determining which strand becomes the guide strand depends on the thermodynamic stability of the 5′end. Generally, the strand which has a more unstable 5′end is selected as the guide strand (Khvorova et al., 2003). Another factor in strand selection is the identity of the nucleotide at position 1. AGO proteins tend to have a preference for U nucleotides in this position (Hu et al., 2009). Following selection, the passenger strand can be released and degraded. Subsequently, the AGO protein and the mature miRNA remain associated and play a role in target mRNA repression at the post-transcriptional level. Here, the mature miRNA guides RISC to complementary target sequences in mRNA, often in the 3′UTR. Complementarity ensues between the seed region of the miRNA and the mRNA target. The seed region consists of seven nucleotides, at positions two to eight from the 5′end of the mature miRNA (Grimson et al., 2007). Upon forming a miRNA-mRNA interaction, the AGO protein often recruits a GW family protein, which associates with AGO and contains a silencing domain that plays a role in recruiting proteins to induce gene silencing (Pfaff et al., 2013). These promote translational inhibition and mRNA decay via deadenylation and/or decapping (Djuranovic et al., 2012) (Figure 1F).

The RISC complex also frequently interacts with various RBPs. DDX6 is an RBP that interacts with AGO to promote deadenylation and decapping (Chu and Rana, 2006; Eulalio et al., 2007). Similarly, many RBPs are involved in interactions with miRNAs while paired with their target mRNA at the 3′UTR (Kakumani, 2022). For instance, Cold shock domain-containing protein E1 (CSDE1) is an RBP that competes for binding with AGO2 on a variety of mRNA targets, impeding miRNA-mediated gene silencing (Kakumani, 2022). Furthermore, beyond RBPs with well-known RNA binding domains, intrinsically disordered regions of proteins have been found to interact with RNA. One example is the interaction between the intrinsically disordered protein, Fragile X Messenger Ribonucleoprotein 1 (FMRP) and the miRNA machinery (Basu and Bahadur, 2016). FMRP is involved in the translational regulation of specific mRNA. It is expected that FMRP binds specific mRNA and facilitates interactions between miRNAs and the mRNA to promote translational repression through FMRP interactions with AGO1 (Jin et al., 2004a; Jin et al., 2004b). It has also been shown that FMRP interacts with Dicer, and therefore may be involved in processing miRNA precursors (Jin et al., 2004b).

Mature miRNAs are often transferred to recipient cells via extracellular vesicles (EVs), where they can exert their regulatory roles (Chen et al., 2021). miRNAs are selectively sorted into EVs based on their sequence and/or structure, through the function of several RBPs (Groot and Lee, 2020). RPBs target specific miRNAs in a sequence-dependent manner for loading into EVs. One such example is synaptotagmin-binding cytoplasmic RNA-interacting protein (SYNCRIP), also known as hnRNPQ (Santangelo et al., 2016). SYNCRIP directly binds miR-3470a and miR-194-2, which contain the hEXO motif (GGCU) responsible for their recruitment to EVs. Interestingly, SYNCRIP was also shown to promote the nuclear processing of pri-let-7a via binding a UAGAAU motif in the apical loop of pri-let-7a and interaction with DGCR8 (Figure 2L) (Chen et al., 2020). Another hnRNP involved in miRNA sorting into EVs is hnRNPA2B1. hnRNPA2B1 must be SUMOylated to interact with GAGG sequences in miR-198 for recruitment to EVs (Villarroya-Beltri et al., 2013). Furthermore, Connexin43 (Cx43) was shown to be important for miRNA selection and formation of EVs (Martins‐Marques et al., 2022). Cx43 binds specific miRNAs (including miR-133b) or hnRNPs involved in sorting, to selectively sort miRNAs into EVs. Cx43 also forms channels at the surface of EVs, modulating the release of miRNAs into recipient cells with implications in the regulation of various cellular pathways (Martins‐Marques et al., 2022).

## 7 Non-canonical miRNA biogenesis

Although it was once believed that most miRNAs are processed through the canonical miRNA biogenesis pathway, research in recent years has highlighted several examples whereby miRNAs are processed through a non-canonical mechanism. These pathways may exclude processing by the microprocessor, or in rare cases, Dicer.

**FIGURE 5:**
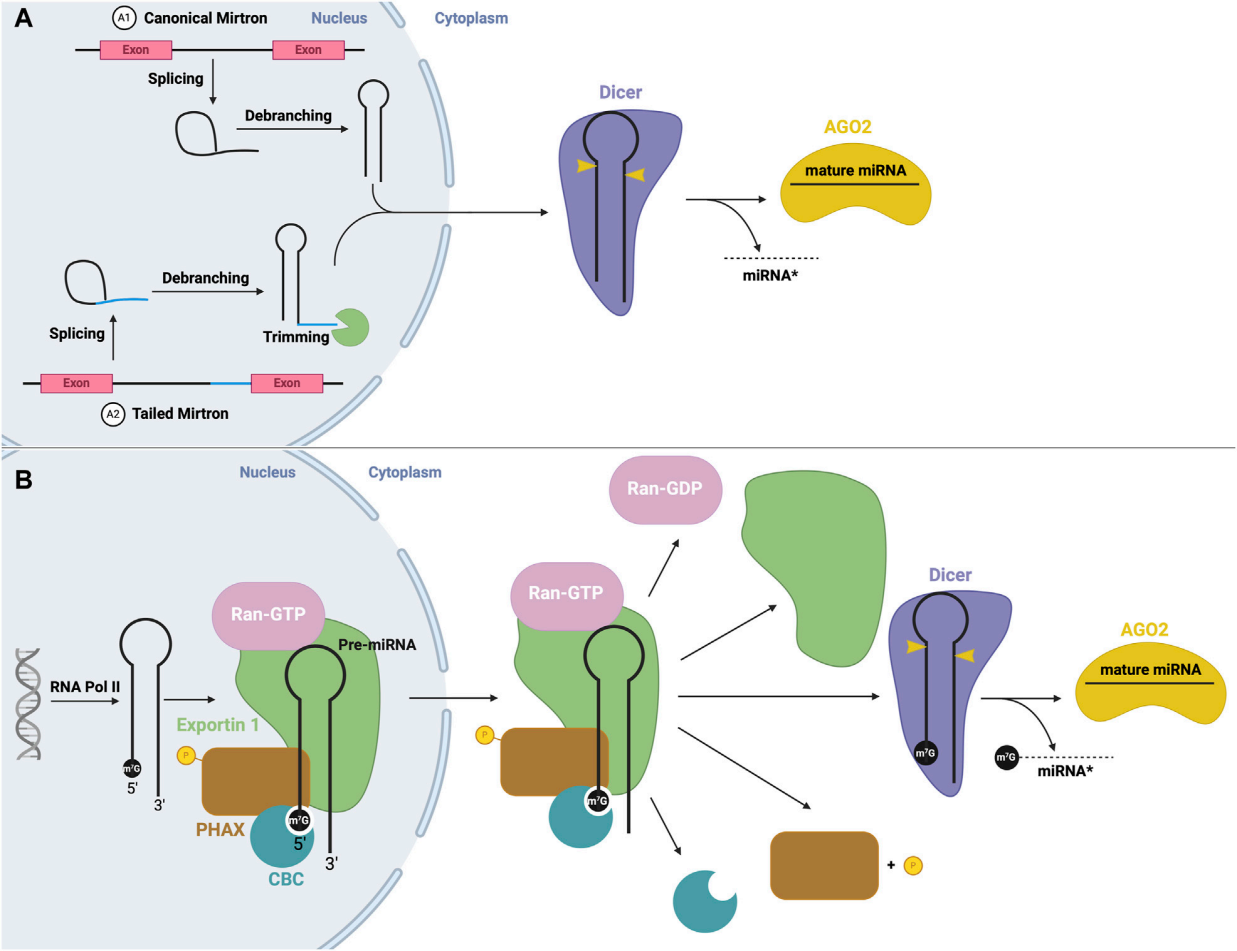
Non-canonical mirtron and modified miRNA biogenesis **(A)** Mirtron biogenesis. mRNA splicing produces an intron lariat, which undergoes debranching, producing a stem-loop structure. Canonical mirtrons (A1) are directly exported to the cytoplasm, and subject to canonical Dicer processing. Tailed mirtrons (A2) must be trimmed before export. **(B)** m7G cap-dependent miRNA biogenesis. Transcription via RNA Pol II produces a short hairpin with a m7G cap, which is recognized by CBC, and forms an export complex with phosphorylated PHAX, exportin one and RAN-GTP. Following nuclear export, PHAX becomes dephosphorylated and GTP hydrolysis occurs, leading to disassembly of the export complex. The hairpin structure is then subject to canonical Dicer processing.

One example of microprocessor-independent processing includes mirtrons. Here, mRNA splicing creates a small RNA from an intron lariat (Okamura et al., 2007; Ruby et al., 2007). Once debranched by a debranching enzyme, the intron folds into a stem-loop structure with a resemblance to pre-miRNA, and thus can bypass cleavage by Drosha (Figure 5A). Conventional mirtrons have both the 5′and 3′ends defined by splicing (e.g., miR-6807) (Ladewig et al., 2012). Alternatively, tailed mirtrons have excess sequences at the 3′or 5′end that require nucleases to trim (Flynt et al., 2010; Westholm and Lai, 2011), known as 3′tailed (e.g., miR-4745) and 5′tailed (e.g., miR-6514) mirtrons (Ladewig et al., 2012). Now, these small miRNAs can be directly exported to the cytoplasm by Exportin 5 (Salim et al., 2022) and undergo Dicer-mediated processing. Additionally, as previously mentioned, microprocessor-independent miRNAs may also arise from the 5′end of transcribed genes, in which transcription terminates early and the transcript folds into a hairpin. This structure contains a m7G cap which is recognized by Exportin 1, by which it is exported to the cytoplasm for processing by Dicer (Xie et al., 2013) (Figure 5B).

**FIGURE 6:**
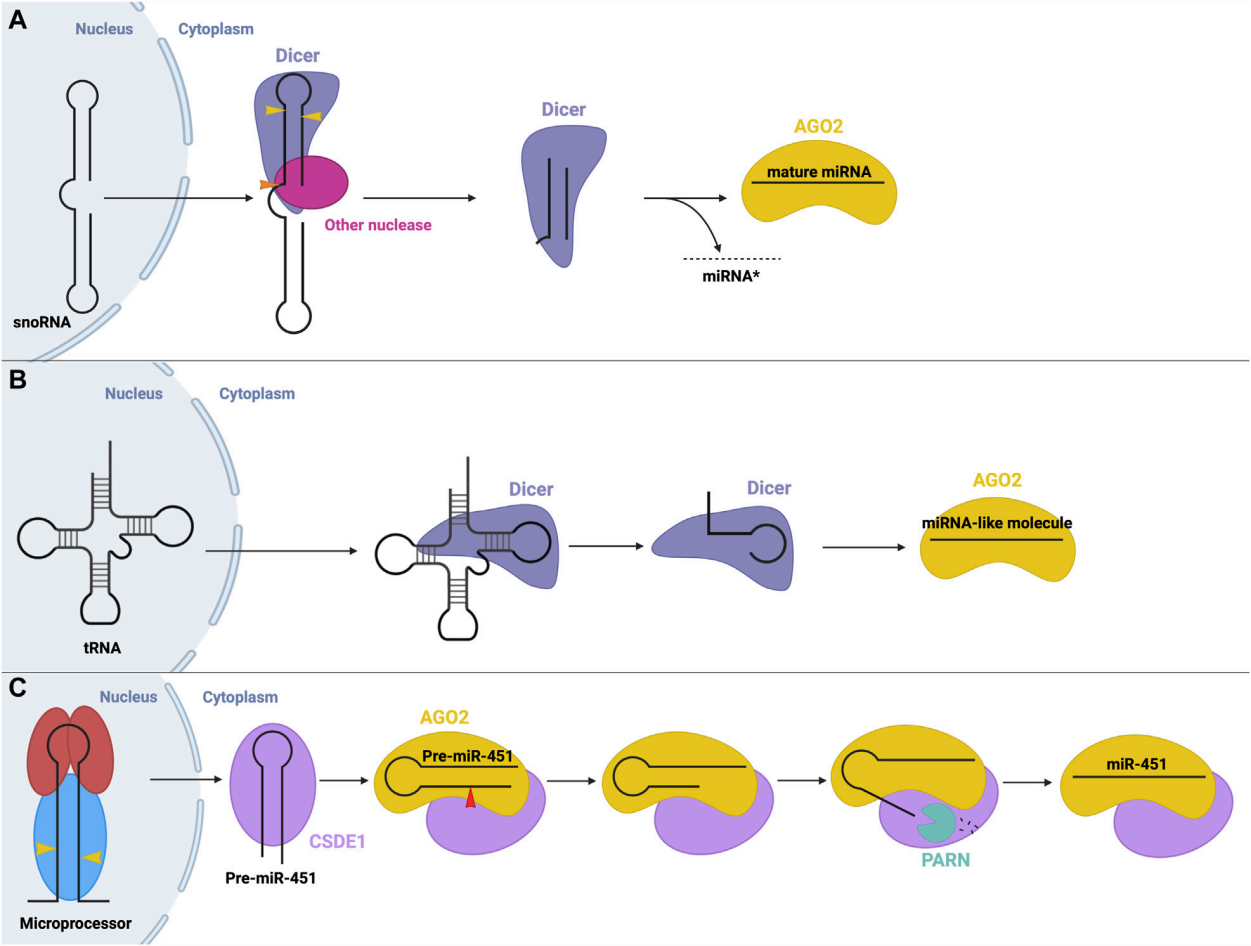
Non-canonical small RNA precursor processing and miRNA biogenesis **(A)** snoRNA precursor biogenesis. A snoRNA precursor is exported from the nucleus to the cytoplasm where it undergoes cleavage by Dicer and possibly other unknown nucleases to produce a miRNA-like molecule that can function in gene silencing. **(B)** tRNA precursor biogenesis. tRNA molecules are exported from the nucleus to the cytoplasm where they can be cleaved into fragments that resemble a miRNA molecule that can function in gene silencing. **(C)** Dicer-independent miRNA biogenesis. The microprocessor generates pre-miR-451 via canonical means. Following export, pre-miR-451 associates with CSDE1 to promote interactions with AGO2, which cleaves the pre-miRNA. A poly(A)-dependent ribonuclease (PARN) trims the 3′end, resulting in mature RISC, whose recruitment is aided through interactions with CSDE1.

Another non-canonical process is the production of miRNA from small nucleolar RNA (snoRNA). In some cases, these snoRNAs are processed by the microprocessor, while others are microprocessor-independent. For example, miRNAs were found to originate from the ACA45 snoRNA, without the function on the microprocessor (Ender et al., 2008). The structure of this snoRNA resembles two miRNA precursors, linked by a hinge. Processing occurs solely through cleavage via Dicer, and potentially other nucleases, in the cytoplasm (Ender et al., 2008) (Figure 6A). Similarly, miRNAs may be derived from endogenous short hairpin RNAs. miR-320 and miR-484 are derived from endogenous short hairpin RNAs independent of the microprocessor complex. The precursors of these miRNAs lack the hairpin flanking sequences required for recognition by the microprocessor (Abdelfattah et al., 2014). The hairpin structure is processed by an unknown mechanism, followed by canonical cleavage by Dicer (Babiarz et al., 2008).

Interestingly, transfer RNAs (tRNAs) may also be a source of miRNA precursors through different microprocessor-independent mechanisms (Stavast and Erkeland, 2019). In one mechanism, Dicer cleaves the tRNA stem into fragments which can be loaded onto AGO proteins to function in post-transcriptional gene silencing similar to miRNAs (Kumar et al., 2014; Li et al., 2018) (Figure 6B).

Although rare, Dicer-independent miRNA biogenesis is also possible. Here, nuclear processing follows the canonical pathway, but in the cytosol, the cleavage step is instead carried out by AGO2 (aka Slicer). AGO2 possesses RNase H-like endonuclease activity which can cleave certain miRNA precursors, such as miR-451 (Figure 6C) (Cheloufi et al., 2010; Cifuentes et al., 2010). Pre-miR-451 generated by Drosha cleavage is too short to be cleaved by Dicer (about 40 nt), so instead directly binds AGO2, which performs the cleavage step. Next, a poly(A)-specific ribonuclease (PARN) is required to trim the 3′ends of the miRNA intermediate (Yoda et al., 2013), resulting in the final, mature miR-451, which remains associated with AGO2 to carry out its gene-suppressing function. CSDE1, an RBP, assists in this process as it binds the UGAU motif in pre-miR-451 and recruits AGO2 and PARN to promote the biogenesis of miR-451 (Kakumani et al., 2023). miR-451 is involved in the regulation of erythropoiesis (Bruchova et al., 2007; Bruchova et al., 2008). Similarly, miR-486, which also plays a regulatory role in erythrocytes, requires AGO2 for its maturation. Biogenesis of miR-486 initially follows the canonical pathway, however, post-Dicer-mediated processing, AGO2 slicer activity is required to cleave and remove the passenger strand, while canonically, the passenger strand dissociates independently (Jee et al., 2018; Treiber et al., 2019).

**FIGURE 7:**
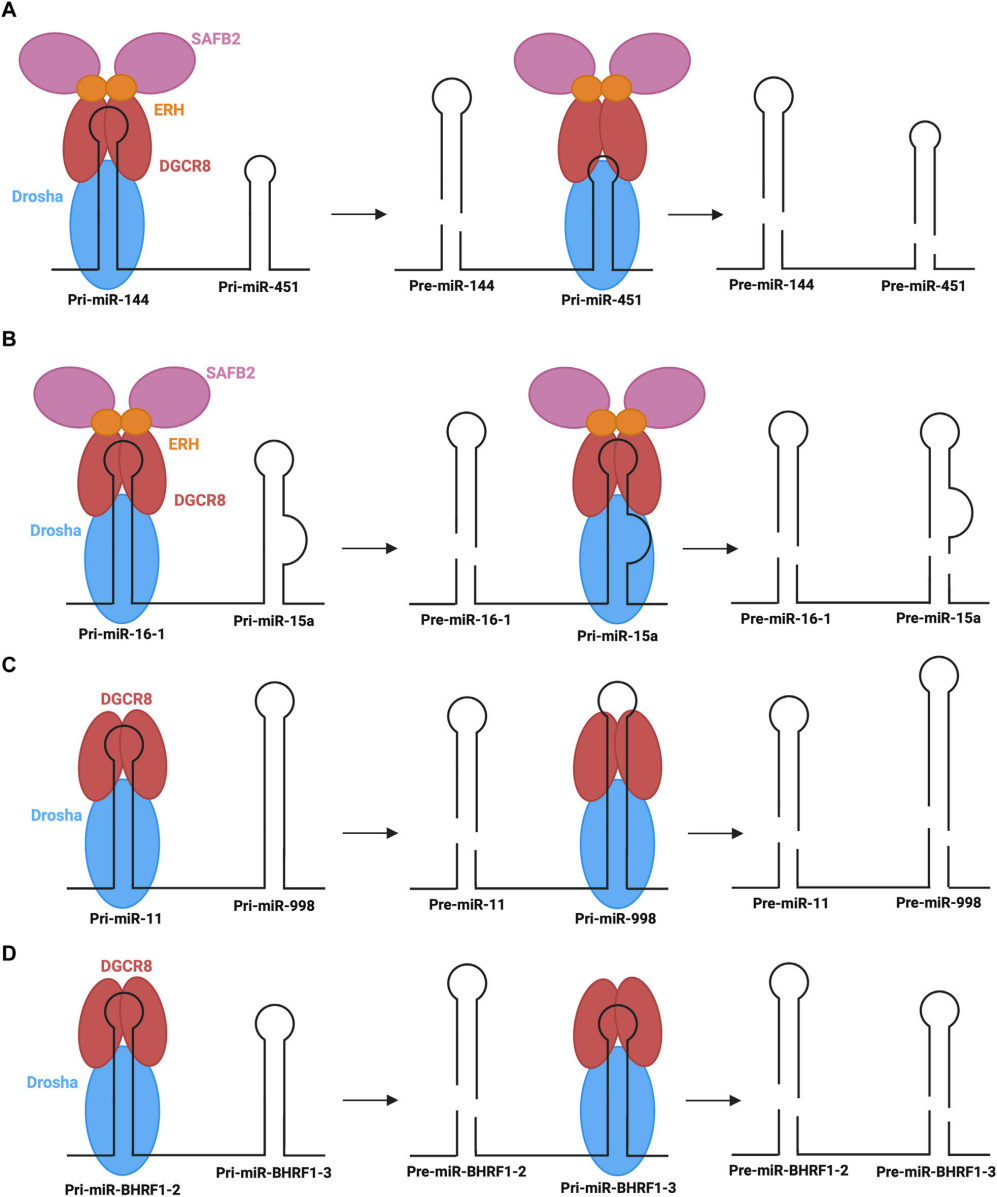
Cluster assistance phenomenon for sub-optimal processing of pri-miRNAs **(A,B)** The processing of pri-miR-451 and pri-miR-15a depends on their transcript neighbours, pri-miR-144 and pri-miR-16-1, which recruit the microprocessor to pri-miR-451 and pri-miR-15a, respectively, with the assistance of SAFB2 and ERH. **(C)** pri-miR-998 processing depends on transcript neighbour, pri-miR-11, which helps recruit the microprocessor to pri-miR-998. **(D)** In Epstein-Barr Virus, pri-miR-BHRF1-3 processing depends on transcript neighbour, pri-miR-BHRF1-2, which helps recruit the microprocessor to pri-miR-BHRF1-3 for the generation of respective pre-miRNAs.

Another emerging non-canonical mechanism is the cluster assistance phenomenon, particularly relevant for sub-optimal processing of pri-miRNAs. Normally, pri-miRNAs contain several structure and sequence features that are essential for recognition by the microprocessor, as described above. When miRNAs, specifically those termed sub-optimal, lack several of these key features, the recruitment and transfer of the microprocessor may be facilitated by a canonical miRNA within the same operon and, thus, the primary transcript (Shang et al., 2020). Approximately 30%–40% of vertebrate miRNAs are found in clusters (of two or more miRNAs) in the genome (Altuvia et al., 2005). Several recent studies suggest that one reason for this conserved clustering may be the involvement in the sub-optimal pri-miRNA processing (Truscott et al., 2016; Fang and Bartel, 2020; Hutter et al., 2020; Shang et al., 2020). The most well-characterized example of this phenomenon is the dependence of miR-451 biogenesis on neighbouring miR-144 (Fang and Bartel, 2020; Shang et al., 2020). As described above, miR-451 is the only identified example of Dicer-independent biogenesis. Although miR-451 is processed via the microprocessor, its sub-optimal structure with abnormally short stem length and small terminal loop presents challenges in nuclear processing efficacy (Fang and Bartel, 2020). In this mechanism, miR-144, which has many optimal pri-miRNA features, recruits the microprocessor to carry out its own biogenesis step, followed by recruiting the microprocessor to pri-miR-451 (Figure 7A). The presence of miR-144 in the same transcript is expected to cause a 40-fold increase in miR-451 (Fang and Bartel, 2020). Interestingly, the identity of the neighbouring miRNA is not significant, provided that it has optimal features for microprocessor recruitment (Shang et al., 2020). Further, less-studied examples of the cluster assistance phenomenon have also been found in humans, *Drosophila*, and viral miRNA clusters. One such example in humans is the biogenesis of miR-15a assisted by the presence of miR-16-1 in the same cluster (Hutter et al., 2020). pri-miR-15a is a weak substrate for the microprocessor due to a relatively large unpaired region within its stem, so the optimal structure of miR-16-1 in the same transcript is critical for the nuclear processing step (Figure 7B). Another recent example is miR-998 processing, dependent on miR-11 in a miRNA cluster in the *Drosophila* E2f1 gene (Truscott et al., 2016). Here, the above-average length of miR-998 makes recognition by the microprocessor difficult, so recruitment is predicted to be improved by the presence of miR-11 within the same transcript (Figure 7C). Interestingly, an additional example of this regulatory process was found in clustered viral miRNAs of the Epstein-Barr virus, whereby miR-BHRF1-3 processing relies on the presence of its neighbour, miR- BHRF1-2 (Figure 7D) (Feederle et al., 2011; Haar et al., 2016).

Although the exact mechanism through which the cluster assistance phenomenon occurs is not known, recent studies have provided insight into potential mechanisms mediated by interactions with accessory proteins (Fang and Bartel, 2020; Hutter et al., 2020). Of particular note are scaffold attachment factor B2 (SAFB2) and enhancer of rudimentary homologue (ERH). A homodimer of ERH interacts with the DGCR8 dimer of the microprocessor (Kwon et al., 2020). Furthermore, ERH can also interact with SAFB2, whereby EHR dimers could link the microprocessor and SAFB2 (Drakouli et al., 2017). Based on these observed interactions, two mechanisms have been proposed to mediate the cluster assistance phenomenon (Fang and Bartel, 2020). Firstly, the microprocessor may initially recognize the optimal hairpin and promote the recruitment of another microprocessor complex to the sub-optimal hairpin through dimer interactions between SAFB2 proteins. Alternatively, a single microprocessor may be recruited to the optimal hairpin, followed by transfer to the suboptimal hairpin with the help of accessory proteins (Figures 7A,B). Currently the former is the preferred mechanism, however further studies are required to elucidate the exact mechanistic insights under endogenous settings.

## 8 Conclusion

miRNAs are critical for normal cellular function due to their vital roles in post-transcriptional gene silencing. miRNAs target a wide variety of mRNAs encoding proteins with diverse cellular functions and thus, it is conceivable that aberrant expression of miRNAs has the potential to disrupt crucial biological processes, such as cell proliferation, and apoptosis and contribute to the development of numerous human diseases. To maintain tight control over miRNA expression and function, they are regulated at multiple levels throughout their biogenesis and functional pathway, via a series of complex interactions between the core catalytic proteins of the pathway and RBPs. Several RBPs make up the miRNA biogenesis machinery which mediates nuclear processing, nuclear export, cytosolic processing, and RISC formation (Table 1). RBPs form RNA-protein and protein-protein interactions with the biogenesis machinery and the AGO protein complexes to fine-tune miRNA expression levels in cells. Therefore, the involvement of RBPs in various steps of miRNA processing and function has been a crucial topic of research in recent years with many promising developments. In particular, recent work by Treiber *et al.* (2017) highlights nearly 180 RBPs that are potentially involved in miRNA biogenesis in different conditions (Treiber et al., 2017). Additionally, Nussbacher *et al.* identified 116 RBPs that potentially regulate miRNAs with cell line specificity through an eCLIP approach (Nussbacher and Yeo, 2018). Their findings highlight a global theme of RBPs playing a regulatory role in the tight regulation of miRNA biogenesis which led to new developments in the field whereby Pradhan *et al.* (2021) used miRNA-RPB interaction networks to predict miRNA expression profiles (Pradhan et al., 2021). However, the full functional characterization and validation of these observed miRNA-RBP interactions in the maintenance of mature miRNA expression profiles remain a challenge and thus incomplete in terms of the regulatory roles of each of the RBPs in specific miRNA biogenesis. Thus, it would be intriguing for future studies to focus on the repertoire of miRNA precursors bound by the core miRNA processing factors, namely, Drosha, Dicer in the absence or the over-expression of each RBP and evaluate how the changes in miRNA binding by both the RBP and the core proteins manifest into the expression levels of specific miRNAs *in vivo*.

**TABLE 1:** Summary of RNA-binding proteins involved in miRNA biogenesis.

RNA-binding protein	Target miRNA(s)	Binding motif in RNA	Stage of biogenesis	Interactions with biogenesis proteins	Effect on miRNA biogenesis	Cell line	References
DDX5 (p68)	Most miRNA		Pri-miRNA cleavage	Microprocessor	Promote	HeLa, 293FT	Shiohama et al. (2007), Beezhold et al. (2010)
DDX17 (p72)	Most miRNA		Pri-miRNA cleavage	Microprocessor	Promote	HeLa, 293FT	Shiohama et al. (2007), Beezhold et al. (2010)
SRSF3		CNNC	Pri-miRNA cleavage		Promote	HEK293	Auyeung et al. (2013)
KSRP	Let-7 family, miR-196a, miR-155	G-rich regions of the terminal loop	Pri-miRNA cleavage and pre-miRNA cleavage	Drosha	Promote	HeLa, U2OS, P19, NIH-3T3, bone marrow derived macrophages, RAW 264.7 macrophages	Ruggiero et al. (2009), Trabucchi et al. (2009), Castilla-Llorente et al. (2013)
hnRNPA1	miR-18a, Let-7a	G-rich regions in the pri-miRNA terminal loop	Pri-miRNA cleavage		Promote or inhibit	HeLa	Guil and Cáceres (2007), Michlewski et al. (2008), Michlewski and Cáceres (2010), Castilla-Llorente et al. (2013)
TDP-43	miR-143, miR-574	GU-rich regions in the terminal loop	Pri-miRNA cleavage and pre-miRNA cleavage	Drosha	Promote	HEK293T, SK-N-BE(2)-C, SH-SY5Y	Kawahara and Mieda-Sato (2012), Di Carlo et al. (2013)
LIN28B	Let-7	Pri-miRNA terminal loop and GGAG in the stem	Pri-miRNA cleavage		Inhibit	HEK293, HeLa, H1299, Igrov1, HepG2, T47D, MDA-MB-231, SK-Mel-28	Heo et al. (2009), Nam et al. (2011), Piskounova et al. (2011), Mayr et al. (2012), Mayr and Heinemann (2013)
HuR/MIS2	miR-7	AUAA in Pri-miRNA terminal loop	Pri-miRNA cleavage		Inhibit	HeLa, human brain astrocytoma 1321N1, HEK293T, SH-SY5Y	Choudhury et al. (2013)
YB-1	miR-29b-2	UYAUC in miRNA terminal loop	Pri-miRNA cleavage and pre-miRNA cleavage		Inhibit	U251-MG	Wu et al. (2015)
Matr3	miR-138-2	Pri-miRNA terminal loop	Pri-miRNA cleavage or nuclear export		Inhibit	E18 Sprague-Dawley rat cells	Weiss et al. (2019)
EWS	miR-34a, miR-122, mir-222, etc.	Pri-miRNA terminal loop flanking regions	Pri-miRNA cleavage		Promote	HeLa	Ouyang et al. (2017)
QKI5	miR-124-1	QKI response element	Pri-miRNA cleavage	DGCR8	Promote	CD34^+^ HSC	Wang et al. (2017)
Exportin 5	Most miRNA	Double stranded pre-miRNA stem and 3’ 2-nt overhang	Nuclear Export		Promote	HeLa	Bohnsack et al. (2004), Lund et al. (2004), Okada et al. (2009)
Exportin 1	miR-320, miR-484	m^7^G cap	Nuclear Export		Promote	HEK293	Xie et al. (2013)
TRBP	Most miRNA	Regions of pre-miRNA stem-loop with tight base pairing	Pre-miRNA cleavage	DExD/H-box helicase domain of Dicer	Promote	HEK293	Haase et al. (2005), Takahashi et al. (2018), Yoshida et al. (2021)
ADAR1	Most miRNA		Pre-miRNA cleavage	DExD/H-box helicase domain of Dicer	Promote	HEK293	Ota et al. (2013)
DAZL	Several miRNAs	GUU	Pre-miRNA cleavage		Promote	hESC line H9, HSF6	Yan et al. (2022)
LIN28A	Let-7	Pre-miRNA terminal loop	Pre-miRNA cleavage		Inhibit	HEK293, HeLa, H1299, Igrov1, HepG2, T47D, MDA-MB-231, SK_Mel_28, HepG2, Huh7, Hep3B	Heo et al. (2008), Heo et al. (2009), Lightfoot et al. (2011), Nam et al. (2011), Piskounova et al. (2011)
FMRP			Processing in the cytoplasm and promotion of translational repression	AGO1, Dicer	Promote	HeLa, human lymphoblastoid cell lines	Jin et al. (2004a), Jin et al. (2004b), Basu and Bahadur (2016)
CSDE1	miR-451	UGAU	Processing in the cytoplasm	AGO2, PARN	Promote	HEK293T, MEL cells	Kakumani et al. (2023)
SYNCRIP	miR-3470a, miR-194-2	hEXO motif (GGCU)	miRNA sorting into EVs	DGCR8	Promote	Murine hepatocyte 3A cells	Santangelo et al. (2016), Chen et al. (2020)
	Let-7a	UAGAAU in pri-miRNA terminal loop	Pri-miRNA cleavage			HEK293	
hnRNPA2B1	miR-198	GAGG	miRNA sorting into EVs		Promote	Human peripheral blood mononuclear cells, HEK293	Villarroya-Beltri et al. (2013)
Cx43	miR-133b		miRNA sorting into EVs		Promote	HEK293, C33a	Martins‐Marques et al. (2022)
SAFB2			Cluster assistance phenomenon		Promote	HEK293T	Hutter et al. (2020)
EHR			Cluster assistance phenomenon	DGCR8	Promote	HEK293T, Expi293F, HEK293E, K562, HCT116	Fang and Bartel (2020), Hutter et al. (2020), Kwon et al. (2020)

Furthermore, miRNA biogenesis now entails far more than the canonical pathway. Recent studies have highlighted different sources of miRNAs including introns, snoRNAs, tRNAs, and other short hairpin RNAs. These structurally distinct precursors undergo unique miRNA processing steps that may bypass certain canonical steps or utilize alternative machinery for cleavage, export and RISC loading. These emerging mechanisms challenge what was previously believed to be true of miRNA biogenesis, highlighting that there are many alternatives to the originally proposed canonical biogenesis process. This has been an interesting and promising area of recent research; however, details of these novel non-canonical mechanisms remain unclear in terms of RBP involvement, as to whether and how RBPs bind different precursors and control their processing to mature miRNAs in different tissue and cell types under physiological and pathological conditions, and it is likely that many processes leading to the biogenesis of non-canonical miRNAs have yet to be discovered.
